# COVID-19 mortality rate determinants in selected Eastern European countries

**DOI:** 10.1186/s12889-022-14567-x

**Published:** 2022-11-16

**Authors:** Marharyta Sobczak, Rafał Pawliczak

**Affiliations:** Department of Immunopathology, Division of Biomedical Science, Faculty of Medicine, Medical University of Lodz, Zeligowskiego st. 7/9, 90-752 Lodz, Poland

**Keywords:** COVID-19, SARS-CoV-2, Coronavirus disease 2019, Mortality, Multivariable regression, Eastern Europe

## Abstract

**Background:**

The COVID-19 pandemic has caused increased mortality worldwide. We noticed a tendency for higher number of deaths in Eastern European countries. Therefore, we decided to investigate whether any common factor that might be responsible for the increased COVID-19 mortality exists.

**Methods:**

In our cross-sectional study, we conducted the correlation and multiple regression analysis using R basing on the data gathered in publicly available databases. In the analysis, we included variables such as: number of deaths, number of new cases, number of hospitalizations, number of ICU (intensive care units) patients, number of vaccinations, number of boosters, number of fully vaccinated individuals, stringency index, number of reported COVID-19 variant cases, and number of flights. Additionally, we analyzed the influence of population density and median age in particular European countries on total number of COVID-19 deaths. Analyzed data represents periods from start of the COVID-19 pandemic in particular Eastern European Countries: Bulgaria, Croatia, Czech Republic, Hungary, Latvia, Lithuania, Poland, Romania, Slovakia and Slovenia, while as the end of the study the day of January 31, 2022 is considered. Results were considered statistically significant at *p* < 0.05.

**Results:**

Our study showed that mortality rate reflects the number of COVID-19 cases (e.g. for Poland was 0.0058, *p* < 0.001), number of hospitalized patients (e.g. for Poland 0.0116, *p* < 0.001), and patients in intensive care (e.g. for Slovakia 0.2326, *p* < 0.001). Stringency index corresponding to level of introduced restrictions and vaccination can affect the mortality rate of COVID-19 in a country-dependent manner: e.g. for Romania 0.0006, *p* < 0.001; whereas in Lithuania − 0.0002, *p* < 0.001. Moreover, occurrence of B.1.1.7 and B.1.617.2 variants increased COVID-19 mortality rates.

**Conclusion:**

Our analysis showed that crucial factor for decreasing mortality is proper healthcare joined by accurate restriction policy. Additionally, our study shows that COVID-19 vaccination proven successful in COVID-19 mortality prevention.

## Background

COVID-19 (coronavirus disease 2019) pandemic has impacted healthcare, governments, financial as well as cultural areas all over the world [1]. The analysis of mortality in 22 countries between 2015 and 2019 years and in 2020 shows that in 2020 716 616 more deaths were notated compared to previous 5 years, from which around 64.5% of deaths were attributed to COVID-19 [2]. Moreover, in the U.S. in period from March 2020 to October 2020 COVID-19 was the second cause of death after heart diseases in people over the age of 85 years, whereas among people aged between 45 and 84 years COVID-19 was the third cause of death [3]. In addition, COVID-19 is usually asymptomatic or causes poor symptoms in children and people younger than 18 years, there is a low rate of death among children and people younger than 18 years due to COVID-19 worldwide [4]. Analysis carried out in England showed that among 61 deceased children and people younger than 18 years with positive SARS-CoV-2 (severe acute respiratory syndrome coronavirus 2) test, 25 died due to COVID-19 in period between March 2020 and February 2021 [4]. Moreover, there are the spatial differences in excess mortality of COVID-19. For example, the study carried out in Italy showed presence of the areas in which higher mortality among men above 75 years old during first pandemic wave and, simultaneously, the lower mortality during the second pandemic wave had been reported. On the other hand, there were areas, in which were lower COVID-19 deaths during first pandemic wave and higher mortality during the second pandemic wave [5]. Interestingly, the cross-section study that analyzed mortality data from 67 countries showed the differences between excess mortality COVID-19 data and COVID-19 confirmed mortality data in some countries [6]. Similarly, COVID-19 deaths and percentage of excess mortality of COVID-19 were varied by provinces in Canada, which can be result in different responses pandemic in different provinces as well as different data reporting systems [7]. Moreover, large systematic analysis of COVID-19 deaths from 74 countries and 266 subnational locations showed the differences between estimated and reported COVID-19 mortality at more than 3 times. Among analyzed countries in our study, the highest differences was reported in Latvia and Lithuania (2.72 and 2.7 times, respectively); while the lowest differences were in Slovakia and Slovenia (1.53 and 1.25, respectively) [8]. According to WHO (World Health Organization) Coronavirus (COVID-19) Dashboard [9], from the beginning of the pandemic until July 28, 2022, approximately 6.4 million deaths have been reported. Of these deaths, more than 2 million are confirmed in Europe.

We noticed that among the economically moderately developed countries in Europe there are countries with a very high rate of death due to COVID-19. Therefore, in our cross-sectional study, we decided to investigate whether there are common factors that influenced increased COVID-19-related mortality rate between ten selected Eastern European countries characterized by similar level of economic development according to GDP (Gross Domestic Product) per capita values such as Bulgaria, Croatia, Czech Republic, Hungary, Latvia, Lithuania, Poland, Romania, Slovakia and Slovenia.

## Methods

### Data search and extraction

The first step of our cross-sectional study was selection of ten Eastern European countries for analysis. Therefore, we calculated the numbers of total deaths related to COVID-19 per million, which was calculated using numbers of total deaths from *WHO Coronavirus (COVID-19) Dashboard* [9] and numbers of population from *Worldometer* [10] as of February 1, 2022. We selected ten European countries with similar economic development and the highest numbers of deaths due to COVID-19: Bulgaria, Croatia, Czech Republic, Hungary, Latvia, Lithuania, Poland, Romania, Slovakia and Slovenia. Next, for our analysis we used data obtained from databases, such as *Our World in data* [11] and *European Centre for Disease Prevention and Control* [12] in period from the start of COVID-19 pandemic and before January 31, 2022. We extracted data, such as:

- new deaths per million that means daily numbers of deaths due to COVID-19 per million people,
- new cases per million that means daily numbers of cases due to COVID-19 per million people,
- hospitalization per million that means daily numbers of hospitalization patients with COVID-19 per million people,
- ICU patients per million that means daily numbers of patients with COVID-19 in intensive care units per million people,
- new vaccinations smoothed per million that means daily numbers of COVID-19 vaccinations per million people with 7 days smoothed,
- total boosters per hundred that means total numbers of COVID-19 boosters per hundred people,
- people fully vaccinated per hundred that means total numbers of people with all doses of COVID-19 vaccines,
- stringency index that means government response stringency index in scale between 0 and 100,
- numbers of COVID-19 cases with popular SARS-CoV-2 variants: B.1.1.7, B.1.351, B.1.651.2 and B.1.1.529.

Additionally, we extracted data about numbers of flights from *Eurocontrol* [13].

### Outcomes

The primary outcome of this study was to determine the correlation between COVID-19-related mortality – the dependent variable, and number of independent variables using multivariable regression analysis. Following independent variables were selected: new cases per million, hospitalization per million, ICU (intensive care units) patients per million, new vaccinations smoothed per million, total boosters per hundred, people fully vaccinated per hundred, stringency index and numbers of flights. The secondary outcome was dependence between numbers of infected cases with different SARS-CoV-2 variants: B.1.1.7, B.1.351, B.1.651.2 and B.1.1.529 and value of COVID-19 mortality. Additionally, we analyzed the influence of population density and median age in particular European countries on total number of COVID-19 deaths in period from beginning of COVID-19 pandemic and as of January 31, 2022.

### Statistical analysis

For primary outcome, we prepared correlation and multivariable regression analysis for each of selected European countries using raw data as numbers per million or per hundred, except variables such as stringency index and number of flights. For secondary outcome, we used raw data of weekly numbers of cases with SARS-CoV-2 variants that was calculated per million. Moreover, we used total numbers of COVID-19 deaths per million and population density and median age. We considered 0 as missing data. In multivariable regression analysis, we removed independent variables that were not statistically significant until all independent variables were statistically significant. The statistical analysis was prepared using R version 4.0.5. Results were considered statistically significant at p < 0.05.

## Results

### The possible rationale behind the higher mortality due to COVID-19

**Fig. 1:**
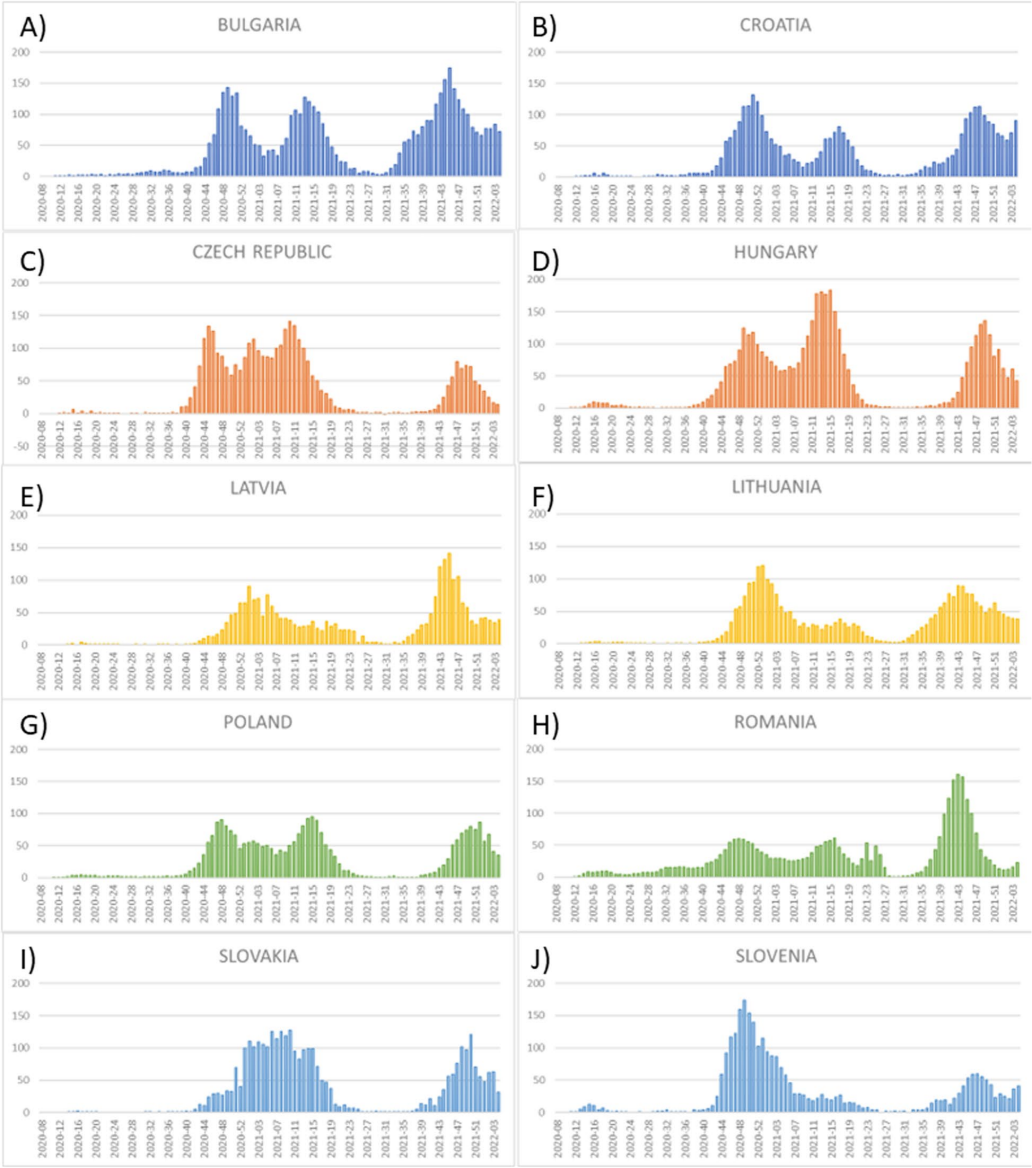
Weekly deaths per million related to COVID-19 in selected ten European countries

**Fig. 2:**
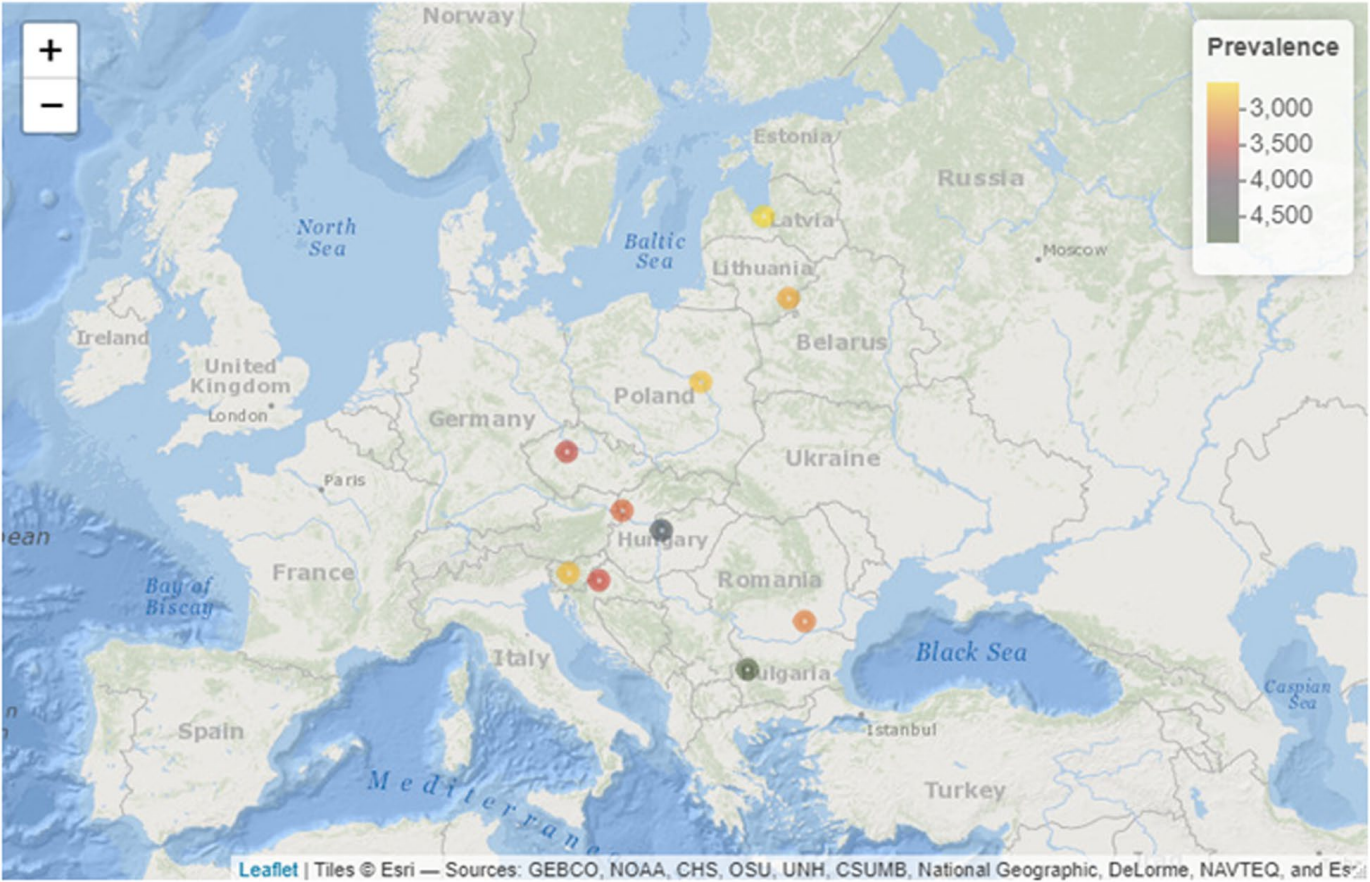
Total deaths per million related to COVID-19 in selected ten European countries

We selected ten countries with similar economic development in the European Union with greater numbers of deaths due to COVID-19: Bulgaria, Croatia, Czech Republic, Hungary, Latvia, Lithuania, Poland, Romania, Slovakia and Slovenia. Figure 1A-J shows weekly numbers of COVID-19 deaths in selected countries from beginning of COVID-19 pandemic and before January 31, 2022. There were two peaks of COVID-19 mortality: between 41st week of 2020 and 21st week of 2021, as well as between 36 week of 2021 and 2 week of 2022. Moreover, Fig. 2 shows that among selected countries Bulgaria and Hungary had the highest rate of COVID-19 mortality.

**Fig. 3:**
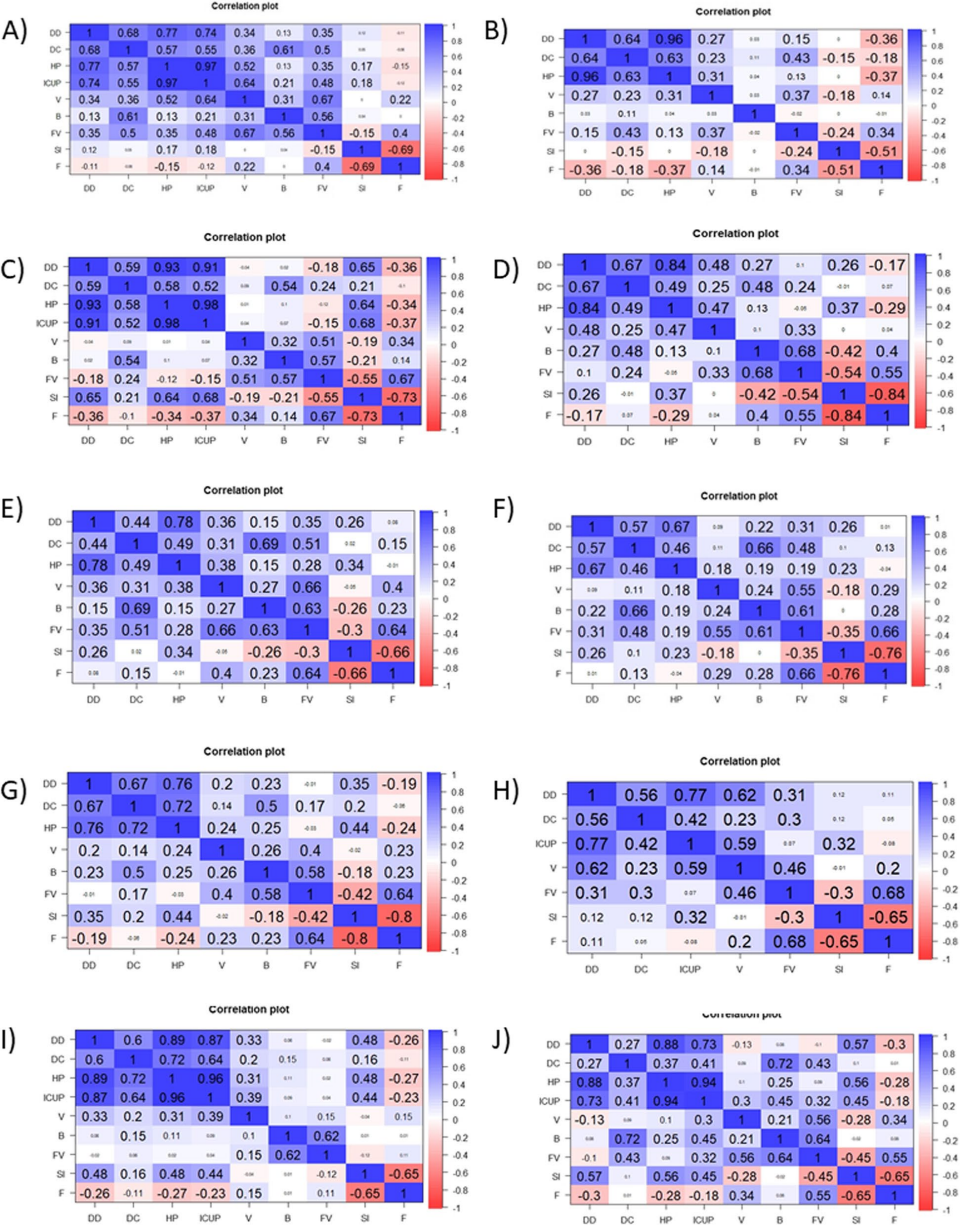
Correlation of number of deaths with analyzed independent variables in selected European countries. **A** Bulgaria, **B** Croatia, **C** Czech Republic, **D** Hungary, **E** Latvia, **F** Lithuania, **G** Poland, **H** Romania, **I** Slovakia, **J** Slovenia. DD – new deaths per million; DC – new cases per million; HP – hospitalization patients per million; ICUP – patients in ICU per million; V – new vaccination smoothed per million; B – total boosters per hundred; FV – people fully vaccinated per hundred; SI – stringency index; F – number of flights

At first, to analyze the reasons of higher mortality in selected countries, we prepared correlation analysis to check the correlation between independent and dependent variables (Fig. 3A-J). As dependent variable daily numbers of deaths per million was used. We prepared two analyses on data from Bulgaria, Czech Republic, Slovakia and Slovenia, because of high correlation between variables, such as number of hospitalized patients per million and patients in ICU per million, as well as on data from Hungary, because of high correlation between stringency index and numbers of flights. Our regression analysis shows (Table 1), that daily number of cases, number of hospitalizations and patients in ICU correlated with increased number of deaths due to COVID-19. On the other hand, vaccinations, booster vaccinations and numbers of fully vaccinated people decreased mortality rate. However, in some countries for example in Bulgaria, Latvia, Lithuania, Romania numbers of fully vaccinated people increased the mortality rate, whereas in Hungary, Slovakia and Romania number of vaccinations and boosters also increased the mortality of COVID-19. Interestingly, stringency index decreased the COVID-19 deaths in Romania, whereas increased in Bulgaria, Czech Republic, Hungary, Lithuania, Poland and Slovakia. The numbers of flights also increased the COVID-19 mortality in Lithuania and Slovenia.

**Table 1:** Results of multiple regression analysis

Country	Intercept	New cases per million	Hospitalization patients per million	ICU patients per million	New vaccination smoothed per million	Total boosters per hundred	People fully vaccinated per hundred	Stringency index	Number of flights	R^2^
Bulgaria	-2.3279**	0.017***	0.0095***	-	-0.001***	-1.7975***	0.15***	0.0372*	ns	0.7368
	-0.5155*	0.0185***	-	0.119***	-0.0015***	-1.9244***	0.0917***	ns	ns	0.7422
Croatia	-0.6299***	0.0007***	0.0232***	NA	-0.00008**	ns	ns	ns	ns	0.9225
Czech Republic	-1.361***	0.0021***	0.0188***	-	ns	-0.1358***	ns	0.0276***	ns	0.8837
	-0.0074	0.0029***	-	0.1057***	-0.00009**	-0.1589***	-0.0149***	ns	ns	0.8721
Hungary	-1.911***	0.0061***	0.0153***	NA	0.0003***	0.0508*	ns	0.0263**	-	0.8094
	-0.6212**	0.0064***	0.016***	NA	0.0002***	ns	ns	-	ns	0.8066
Latvia	-0.7138***	0.0009*	0.0184***	NA	ns	-0.1791***	0.045***	ns	ns	0.6385
Lithuania	-3.393***	0.0034***	0.0085***	NA	-0.0002***	-0.2093***	0.057***	0.0735***	0.013*	0.6114
Poland	-0.8195	0.0058***	0.0116***	NA	ns	ns	ns	0.017*	ns	0.6109
Romania	0.313	0.0057***	NA	0.1001***	0.0006***	NA	0.0288**	-0.0229**	ns	0.7212
Slovakia	-1.734***	ns	0.022***	-	0.0002***	ns	-0.0286*	0.0262***	ns	0.8048
	-2.1833***	0.0011***	-	0.2326***	ns	ns	-0.0287*	0.0467***	ns	0.7804
Slovenia	-0.5043*	ns	0.0267***	-	-0.0005***	-0.096***	ns	ns	0.0106*	0.83
	-0.4357	0.0014***	-	0.14***	-0.0008***	-0.3073***	-0.0365***	ns	0.0211**	0.7492

*NA* not applicable; *ns* not significant; *p < 0.05; **p < 0.01; ***p < 0.001

### The influence of SARS-CoV-2 variants on COVID-19 mortality

**Fig. 4:**
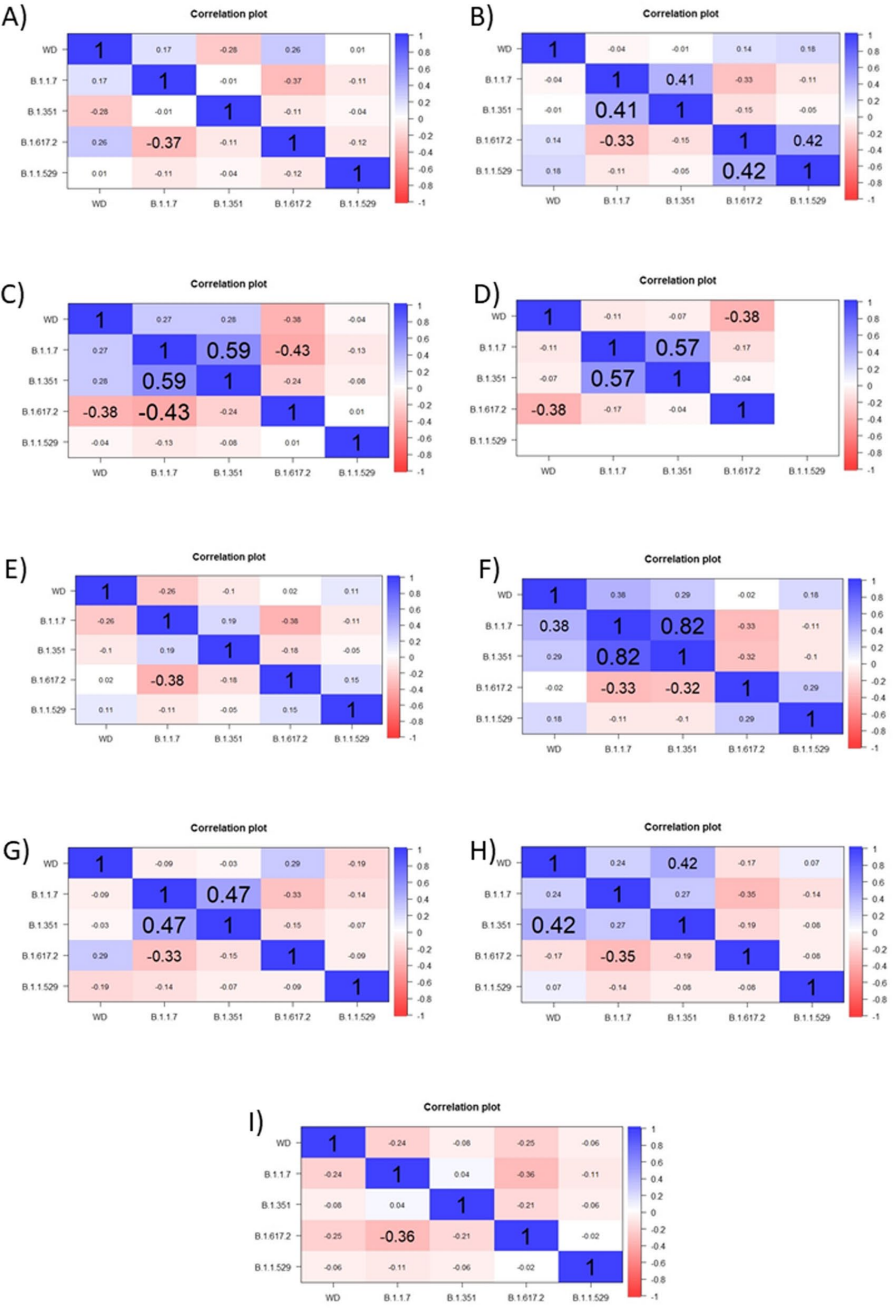
Weekly deaths and selected variants of SARS-CoV-2: B.1.1.7, B.1.351, B.1.617.2 and B.1.1.529. Cumulative plots represent numbers per million. **A** Bulgaria, **B** Croatia, **C** Czech Republic, **D** Latvia, **E** Lithuania, **F** Poland, **G** Romania, **H** Slovakia, **I** Slovenia

**Fig. 5:**
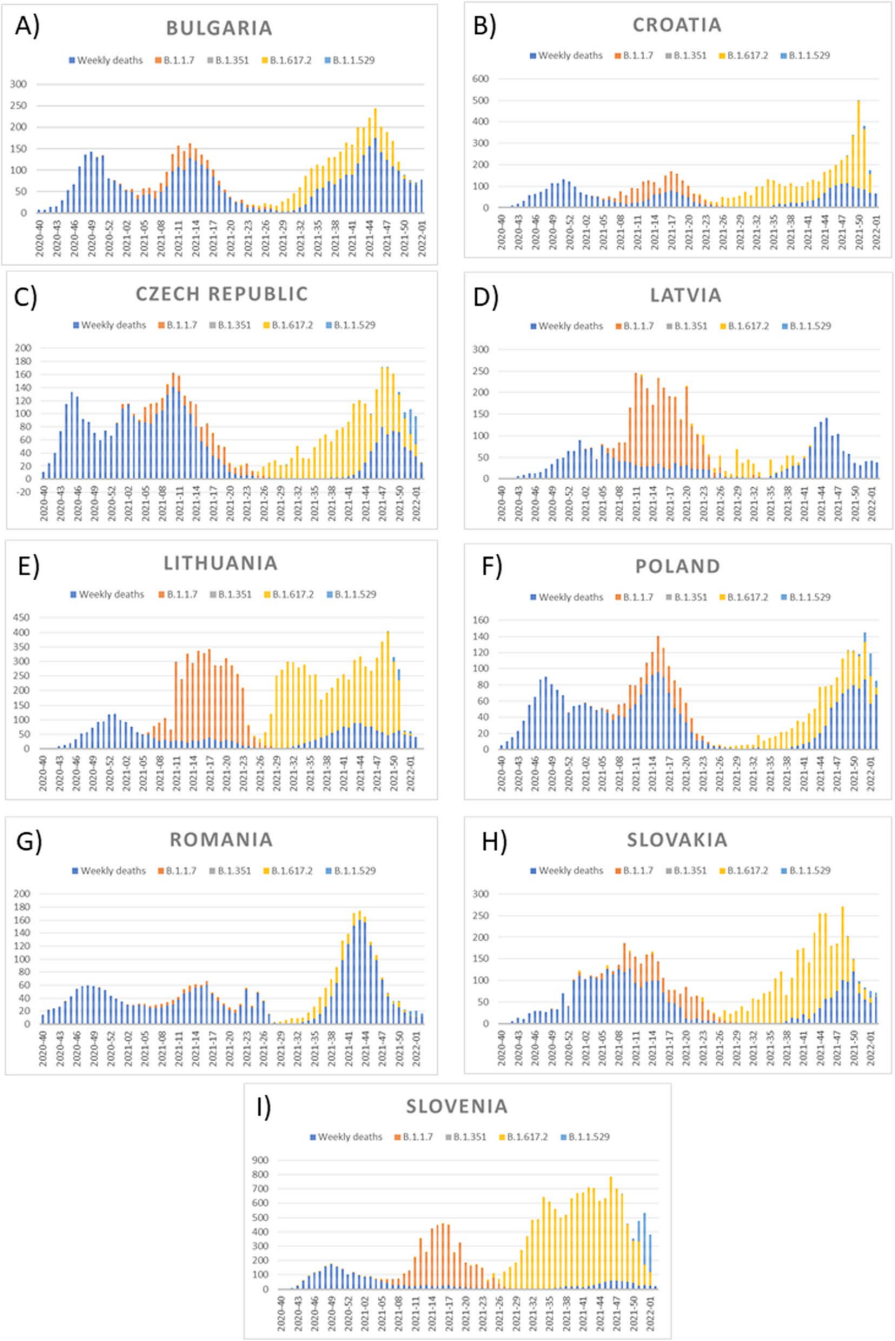
Correlation of deaths with selected variants of SARS-CoV-2 in European countries

We checked, if selected SARS-CoV-2 variants affected on mortality caused by COVID-19, but only on 9 countries, because of missing data from Hungary. Correlation analysis showed that there were weak correlations between popular SARS-CoV-2 variants, such as B.1.1.7, B.1.351, B.1.617.2 and B.1.1.529, and COVID-19 mortality (Fig. 4A-I). However, we observed the positive interdependence between number of patients with B.1.1.7 and B.1.617.2 variants and growth of mortality rate caused by COVID-19 (Fig. 5A-I).

### The influence of population density and median age on COVID-19 mortality

**Fig. 6:**
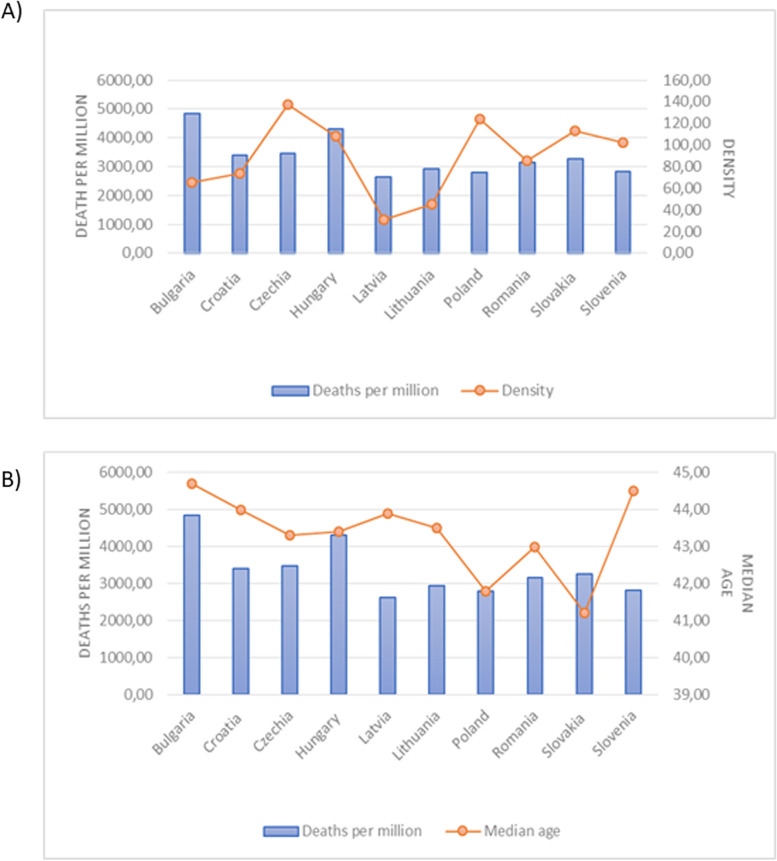
The influence of population density and median age on COVID-19 mortality. **A** population density, **B** median age

Additionally, we checked the effect of population density and median age on numbers of deaths caused by COVID-19. We didn’t observe the dependence between population density (Fig. 6A) and median age (Fig. 6B) and COVID-19 mortality rate.

## Discussion

In our cross-sectional study, we analyzed the potential reasons of the greater mortality rate caused by COVID-19 in selected Eastern European countries: Bulgaria, Croatia, Czech Republic, Hungary, Latvia, Lithuania, Poland, Romania, Slovakia and Slovenia. Our multivariable regression analysis showed that numbers of COVID-19 cases, numbers of hospitalization patients as well as patients in ICU influenced on the increase of COVID-19 mortality. Despite the fact that we detected weak correlation between B.1.1.7, B.1.351, B.1.617.2 and B.1.1.529 SARS-CoV-2 variants and COVID-19 morality, we observed the tendency with B.1.1.7 and B.1.617.2 variances and deaths due to COVID-19. Up to date, multiple variants of SARS-CoV-2 have been detected. B.1.1.7, B.1.351, B.1.617.2 and B.1.1.529 variants are considered more dangerous in terms of severity than wild-type virus, whereas B.1.351 and B.1.617.2 tend to give more severe symptoms of all COVID-19 variants [14]. According to other studies, prevalence of particular COVID-19 variants affects mortality rates of the virus [15, 16].

In order to limit spread of COVID-19 pandemic, numerous countries decided to implement number of preventive measures such as mandatory mask wearing, social distancing, travel restrictions, remote working. All of above measures potently restricted spread of coronavirus in the long run, when introduced simultaneously [17]. These restrictions, were accompanied by spreading awareness about relevance of proper hygiene, such as hand washing. Due to the fact numerous hand washing and sanitizing facilities were publicly dispensed. Moreover, governments encouraged sanitizing of publicly available areas such as public transport vehicles or classrooms. Although arousing many controversies, and leading to potential economic losses lockdowns were introduced in many countries [18]. However, our analysis showed that stringency index, which means scaled response of government, can decrease the COVID-19 mortality rate only in Romania, whereas can increase in Bulgaria, Czech Republic, Hungary, Lithuania, Poland and Slovakia. These positive correlations can be explained by fact, that COVID-19 restrictions were introduced in the same period of time as number of COVID-19 cases increased, which in turn is reflected by COVID-19 deaths. Megarbane et al. [19] in their study basing on data published by WHO assumed that early introduction of lockdown potently hampered spread of the virus as well as possibly shortened the pandemic. They propose, that lockdowns should be considered as a standard counter-measure to future epidemics as well. Moreover, another study conducted on COVID-19 mortality data pointed out that COVID-19-related mortality rates values varied among 40 investigated European countries. The study suggested that the crucial factor affecting the number of deceased from COVID-19 was the time required for governments to ban public events [20].

Bhowmik et al. [21] in their study reported that COVID-19 transmission strongly related on mobility of subjects measured by daily exposure and percentage of people staying at home. Another study, concluded that due to mobility reduction in the analyzed regions of Italy, approximately 4793 deaths were avoided in given period of time [22]. In our analysis, we decided to check the influence of mobility, represented by number of flights, as these may be easier monitored and reported than mobility of particular individuals, on COVID-19-related mortality. Our results, are partially in line with described data described in aforementioned studies as for Lithuania, Slovakia and Slovenia, we observe a statistically significant trend, that increased mobility is correlated with higher COVID-19 death-count.

As the vaccination was the most promising anti-COVID-19-related mortality measure, the big portion of world attention was paid to the topic. According to the numerous studies the vaccination rate vastly affects mortality rates in COVID-19 patients. Interestingly, in our study as numbers of distributed vaccine doses, booster vaccine doses as well as count of fully vaccinated people rose, mortality of COVID-19 decrease. However, in some countries we got the opposite results: in Bulgaria, Latvia, Lithuania, Romania numbers of fully vaccinated people positively correlated with mortality rate, whereas in Hungary, Romania and Slovakia numbers of vaccine doses and boosters also increased the mortality of COVID-19. We observed that in Bulgaria and Romania the COVID-19 death tide during the Autumn of 2021 was stronger than before, even though the number of fully vaccinated people was also increasing. In addition, Bulgaria has a low percentage of fully vaccinated people. According to COVID-19 Vaccine Tracker [23] from European Centre for Disease Prevention and Control only 29.9% of population was fully vaccinated on day July 21, 2022. Moreover, we observed a trend that the daily number of vaccinations per million coincided with the number of daily deaths per million in Romania, Hungary and Slovakia. According to the study by Walkowiak et al. [24], in most EU countries vaccination rate reaches 75%, except Eastern Bloc countries. In case of Eastern Bloc, the value is much lower, whereas the least can be observed in Bulgaria and Romania. By comparative analysis of vaccination policy in Poland and Lithuania, authors assumed that introduction of vaccine certificates, and extensive restrictions for unvaccinated citizens successfully increased the vaccination rate in Lithuania. Besides the vaccination policy, proper health-related education seem to be relevant in terms of vaccination ratio, as people not educated in medical sciences tended to be less likely to be vaccinated than people with a medical background. Additionally, an important factor related to lower vaccination rate in at least some of Eastern European countries might be related with relatively high percent of citizens living in secluded communities such as Roma community. Even before the COVID-19 pandemic, Roma community members strayed from vaccine administration as they had impaired access to the healthcare. The issue was extensively investigated by Sandor et al. [25], as they investigated the vaccination ratio among members of secluded communities in Hungary. As people living in these segregated colonies tend to avoid vaccination, the authors highlight urgent need to change governmental vaccination policies in order to persuade their citizens to be vaccinated in order to decrease the COVID-19-related mortality among them. Nevertheless, the main measure that changed COVID-19 pandemic was vaccination, which prevented deaths of millions people. Based on reported COVID-19 deaths, global analysis estimated that first year of COVID-19 vaccination prevented about 14.4 million of COVID-19 deaths, whereas according to excess mortality data, vaccination prevented 19.8 million of deaths [26].

Another determinant of COVID-19 infection spread is density of population. However, we didn’t observe the dependence between population density and mortality caused by COVID-19. Study conducted in the U.S. showed that at the beginning of COVID-19 pandemics, density of population in particular counties was a major factor for the viral infectivity [27]. Of note, that more urbanized areas, characterized by lower percent of green space tend to be characterized by increased infectivity [28]. Nonetheless, density alone seem not to be a crucial factor as some studies point out that increased mortality does not affect countries with better socioeconomic conditions, as it does in case of poorer countries [29]. These observation may be crucial for our analysis as in analyzed countries the socioeconomical level is significantly lower than in e.g. Western Europe. Similarly, we didn’t observe the interaction between median age and COVID-19 mortality rate in analyzed countries, in which median age ranged between 41 and 45 years. COVID-19 mortality depends on age was exponentially increased with higher age in 2020 [30]. However, the analysis from U.S. showed that COVID-19 mortality decreased by a higher percentage at older ages than at younger ages in period from July 2021 to October 2021 [31].

Higher COVID-19 mortality in Central and Eastern European countries compared to Western Europe might be also caused by the overall healthcare quality. According to the data published by Eurostat, Central/Eastern European countries tend to spend less percent of their budgets on healthcare compared to Western countries [32]. Moreover, many of those countries had to deal with multiple reforms of the healthcare system after fall of USSR (Union of Soviet Socialist Republics), which for economically weaker countries might be a problem up to these days [33].

Unfortunately, our study has several limitations. At first, not all of the data in the databases was complete, which may have affected the results of our analysis. The problem with collecting data on COVID-19 mortality may be related to the varying definition of COVID-19 caused deaths in different countries. Also, as we mentioned earlier, there is a difference between estimated and reported COVID-19 mortality, which could have further influenced our analysis. Among analyzed countries in our study, the highest differences were found in Latvia and Lithuania (2.72 and 2.7 times, respectively); while the lowest were in Slovakia and Slovenia (1.53 and 1.25, respectively) [8]. In addition, we performed regression analysis on the raw data and didn’t include comparisons by age and gender. Moreover, some data were gathered within different time periods, e.g. population density and median age in particular countries were from 2020, whereas data about total numbers of COVID-19 death was from January 31, 2022. Despite the abovementioned limitations to our study, it points out the most important factors that affected COVID-19 mortality rate in Eastern Europe.

## Conclusion

These combined together with worse socioeconomical status, often impaired healthcare, and less-effective vaccination policy among Eastern European countries may reflect the difference in COVID-19 mortality rates when compared to the Western Europe. It is vital for Eastern European governments to spread awareness about severity of the pandemic, propose more strict vaccination policy, improve healthcare and focus on proper education of citizens. Society should learn from the current pandemic and draw conclusions in case if another pandemic appears in the future.

## Data Availability

All data generated or analyzed during this study are included in this published article.

## Abbreviations

COVID-19: Coronavirus disease 2019
SARS-CoV-2: Severe acute respiratory syndrome coronavirus 2
ICU: Intensive care units
WHO: World Health Organization
USSR: Union of Soviet Socialist Republics
